# ﻿*Metagentianajiangyouensis*, a new species of *Metagentiana* (Gentianaceae) from Sichuan, China

**DOI:** 10.3897/phytokeys.247.129934

**Published:** 2024-10-11

**Authors:** Hai-Feng Cao, Jie Cai, Yuan Zou, Hong Sun, Fang-Fang Li, An-dong Xiong, Mei-Jun Xu

**Affiliations:** 1 Shanghai Museum of TCM, Shanghai University of Traditional Chinese Medicine, Shanghai 201203, China Shanghai University of Traditional Chinese Medicine Shanghai China; 2 Germplasm Bank of Wild Species, Kunming Institute of Botany, Chinese Academy of Sciences, Kunming, Yunnan, 650201, China Kunming Institute of Botany, Chinese Academy of Sciences Kunming China; 3 Sichuan Changhong Electronic Holdings Group Co., Ltd., Mianyang 621000, Sichuan, China Sichuan Changhong Electronic Holdings Group Co., Ltd. Mianyang China; 4 Sichuan Nanchong Liuhe Group Co., Ltd., Nanchong 637000, Sichuan, China Sichuan Nanchong Liuhe Group Co., Ltd. Nanchong China; 5 Minhang Qiangwei Primary School Affiliated to Shanghai University of Traditional Chinese Medicine, Shanghai 201100, China Minhang Qiangwei Primary School Affiliated to Shanghai University of Traditional Chinese Medicine Shanghai China; 6 Quality Management Department, Jiangxi Fanglian Pharmaceutical Co., Ltd., Nanchang 330000, Jiangxi, China Quality Management Department, Jiangxi Fanglian Pharmaceutical Co., Ltd. Nanchang China; 7 Affiliated Hospital of Jiangxi University of Traditional Chinese Medicine, Nanchang 330006, Jiangxi, China Affiliated Hospital of Jiangxi University of Traditional Chinese Medicine Nanchang China

**Keywords:** Gentianaceae, *Metagentiana*, Morphology, taxonomy

## Abstract

*Metagentianajiangyouensis*, a new species of Gentianaceae from Sichuan, China, is described and illustrated. This new species is similar to *Metagentianavillifera*, but differs by the fact that the plant is glabrous and has 1–4 flowering stems, well-developed basal vegetative rosettes, a pale purple corolla with dark purple spots on the tube and erose or denticulate, non-fringed plicae margins. It also somewhat resembles *M.rhodantha*, but can be easily distinguished by having shorter stems, entire leaf margins, smooth, shorter sepal lobes, spotted corolla tubes, non-fringed plicae and narrowly winged seeds.

## ﻿Introduction

The genus *Metagentiana* T.N.Ho & S.W.Liu in Gentianaceae was separated from *Gentiana* L. by Ho et al. (2002) based on comprehensive morphological and cytological evidence, and fourteen species were listed in this genus. A recent molecular phylogenetic study of subtribe Gentianinae confirmed that *Metagentiana* was monophyletic when excluding two yellow-flowered species, notably *M.souliei* (Franch.) T.N.Ho, S.W.Liu & Shi L.Chen and *M.striata* (Maxim.) T.N.Ho, S.W.Liu & Shi L.Chen, which were transferred to the new genus *Sinogentiana* Adr.Favre & Y.M.Yuan (Favre et al. 2014). Both morphological and molecular evidence indicates that *Metagentiana* has a close relationship with *Sinogentiana*, *Tripterospermum* Blume and *Crawfurdia* Wall. (Ho et al. 2002; Favre et al. 2014). Currently, excluding the new species, *Metagentiana* contains twelve species: Nine of these are restricted to southwestern China; one is relatively widespread in northwestern and central China; one is distributed in eastern Myanmar, and one is endemic to Thailand.

*Metagentiana* can easily be distinguished from *Tripterospermum* and *Crawfurdia* by having ascending to erect, not twining or trailing, stems. It can be distinguished from *Gentiana* by the following features: stem leaves sessile, widely separated, leaf blades broadly ovate to ovate-triangular; flowers bracteate; calyx keeled or winged, plicae asymmetrical, very oblique; stamens unequal in length, apically recurved; style filiform, as long as or longer than the ovary; capsules cylindrical, wingless; seeds triquetrous with three winged edges, rarely wingless or areolate; seed coat minutely to coarsely reticulate (Ho et al. 2002).

*Metagentiana* and *Sinogentiana* are closely related, and their morphological characteristics are similar but flowers of *Metagentiana* are pink, purple or blue and the stem leaves are 0.3–1.5 cm long or up to 3 cm long in *M.rhodantha* (Franch.) T.N.Ho & S.W.Liu, whereas the corolla of *Sinogentiana* is consistently whitish or yellow and the stem leaves are 1–3 cm long. Most species in *Metagentiana* are annual herbs, except for *M.rhodantha* and *M.villifera* (H.W.Li ex T.N.Ho) T.N.Ho & S.W.Liu, which are perennials with extremely short rhizomes and basal rosettes (Favre et al. 2014).

One of the authors (Y. Zou) photographed an unknown species of Gentianaceae on 22 December 2018. After collection and observation of living material, we confirmed that this species belongs to the genus *Metagentiana* because of the large bracts at the bases of the flowers, the keeled and winged calyx-tube, the unequal stamens that are unilaterally curved downwards, and the filiform and long style. Literature study (Ho and Pringle 1995; Ho and Liu 2001; Struwe et al. 2002; Cao et al. 2019; Li et al. 2019; Yang et al. 2020; Favre et al. 2022; Chen et al. 2024) showed that this perennial species is closely similar to *M.villifera* and *M.rhodantha*. But its fewer branches and well-developed rosette leaves, shorter stems, entire leaf margins and smooth, shorter sepal lobes, as well as its corolla tube with dark purple spots and non-fringed plicae margins distinguish it from *M.villifera* and *M.rhodantha*. We confirm that this species is new to science based on its morphological characters and provide a detailed description below.

## ﻿Materials and methods

The type specimens of the new species were collected from Qianyuan Mountain, Hanzeng Town, Jiangyou County, Mianyang City, Sichuan Province, and deposited in the herbaria of CSH, KUN, PE and SMCM (Shanghai Museum of TCM, Shanghai University of TCM). Measurements and description of morphological characters of the new species were based on dried specimens and living plants. Type specimens of *Metagentianavillifera* (holotype: CDBI 0172347!; isotype: SZ!, KUN!, PE!, SM!; topotype: SM!) and specimens of *M.rhodantha*, which is the most closely related to the new species, were consulted online (Chinese Virtual Herbarium, http://www.cvh.ac.cn/) firstly, and then examined from CDBI, KUN, SZ, PE and SM. The line drawings, the description and most of the photographs were based on the type specimens. The conservation status of the new species was evaluated according to the guidelines of the IUCN Red List Categories and Criteria (IUCN 2024).

## ﻿Taxonomic treatment

### ﻿ Metagentiana jiangyouensis

Taxon classificationPlantaeGentianalesGentianaceae

H.F.Cao sp. nov.

9118DF90-71DD-5BFD-9C2F-8F2B45239101

urn:lsid:ipni.org:names:77349955-1

Figs 1 , 2

#### Diagnosis.

*Metagentianajiangyouensis* is similar to *M.villifera* and *M.rhodantha*, but differs from these species by the fact that the plant is completely glabrous and has green flowering stems 8–11 cm long with 6–9 pairs of leaves per stem, well-developed basal vegetative rosettes, glabrous stem leaves with entire margin, spatulate to lanceolate, 1.3–2(–3) mm long calyx lobes with glabrous margins, a pale purple corolla with dark purple spots on the tube, and erose or denticulate, but never fringed plicae margins.

**Figure 1.**
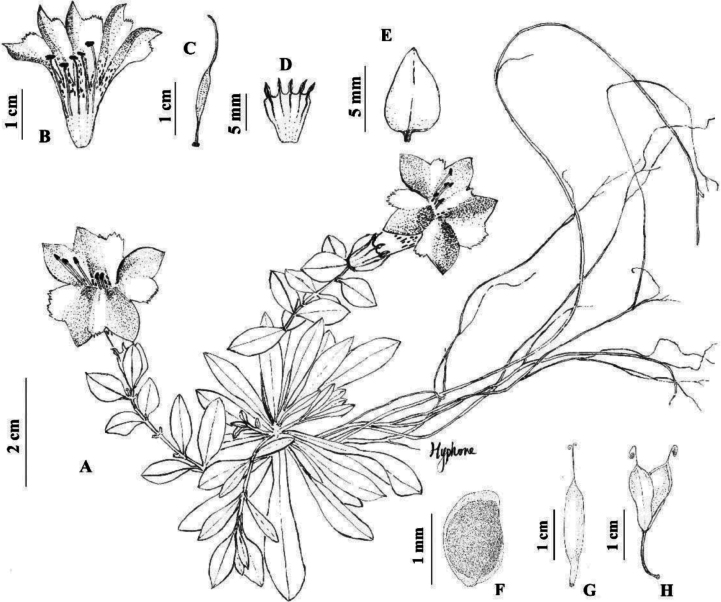
*Metagentianajiangyouensis* H.F. Cao **A** habit **B** longitudinally opened corolla **C** pistil **D** longitudinally opened calyx **E** middle or upper cauline leaf **F** seed **G, H** closed and open fruit. Drawn by H.F. Cao **A–E***Yuan Zou QYS01***F–H***Hai-Feng Cao CAOHF033*.

#### Type.

China • Sichuan: Mianyang City, Jiangyou County, Hanzeng Town, Qianyuan Mountain, on the road to Yinguangdong, growing on a cliff; 31.847981°N, 104.608385°E; 1169 m a.s.l.; 5 January 2019; *Yuan Zou QYS01* (holotype: CSH!; isotype: KUN 1584155!, PE 02402078!, SMCM!).

**Figure 2.**
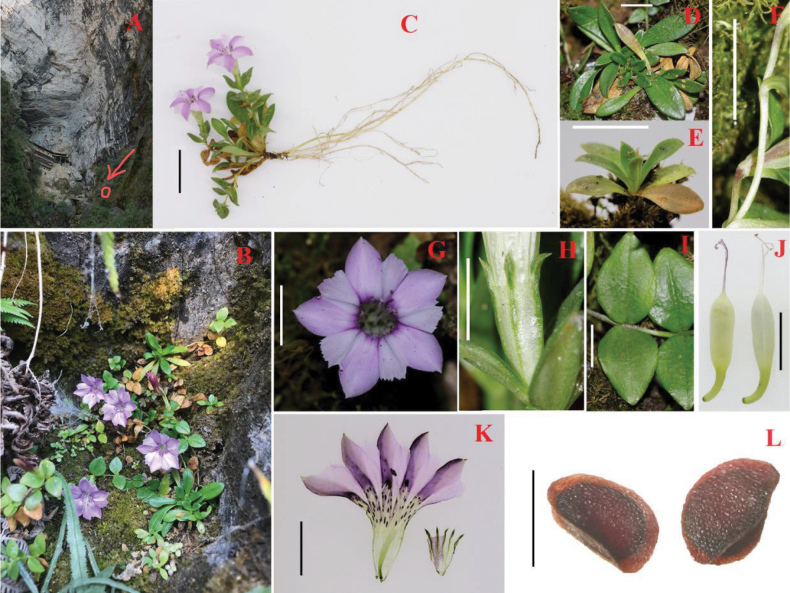
**A, B** habitat **C** habit **D** vegetative rosettes **E** seedling **F** stem **G** corolla, front view **H** calyx, side view **I** cauline leaves, adaxial view **J** fruits **K** opened fresh flower **L** seeds. Scale bars: 2 cm (**C**); 1 cm (**D–G, J, K**); 5 mm (**H, I**); 1 mm (**L**). **A, B** photographed by Y. Zou **C–L** photographed by H.F. Cao **A–I, K***Yuan Zou QYS01***J, L***Hai-Feng Cao CAOHF033*.

#### Description.

Perennial herbs, 8–11 cm tall, with a short rhizome, 5–20 mm long. Flowering branches 1–4, arising from basal rosette, stems green, glabrous, ascending to erect, slender, simple or branched in upper part, 0.5–1.0 mm in diameter when fresh and 0.4–0.6 mm in diameter when dried, each stem with 6–9 pairs of leaves. Basal rosette leaves well-developed and persistent at anthesis; petiole 4–10 mm long, leaf blade oblong-spatulate or elliptic, 10–30 × 2.5–7 mm, both surfaces glabrous, margin entire, apex acute, midvein distinct. Abbreviated vegetative shoots well-developed. Stem leaves widely spaced, shorter than internodes, sessile; blade ovate, elliptic, oblong to oblong-spatulate in basal cauline leaves, and ovate to broadly ovate in middle and upper cauline leaves, 7–12 × 3–7 mm, both surfaces glabrous, margin entire, base rounded, apex acute or obtuse, veins 1–3, base tapering into a short petiole, 0.5–2 mm long. Flowers terminal, solitary, sessile, floral bracts ovate, 7–10 × 4–6 mm, both surfaces glabrous, margin entire, base rounded, apex acute or obtuse, veins 1–3. Calyx tube broadly tubular to tubular-campanulate, glabrous outside, 6–7 mm long, membranous, with 5 prominent green veins but not winged; lobes spatulate to lanceolate, equal or unequal, 1.3–2(–3) × 0.4–0.8(–1.5) mm, base usually slightly shrunken, margin cartilaginous and smooth, apex acuminate, midvein outside prominent, sinus between lobes rounded. Corolla purple, with dark purple spots on corolla tube, 2.9–3.3 cm long, tube funnel-shaped; lobes ovate-triangular, 4.5–6 × 4.5–6 mm, margin entire, apex acute; plicae obliquely truncate or broadly ovate-triangular, 2–3 × 5–6 mm, margin erose or denticulate. Stamens inserted at basal part of corolla tube, unequal; filaments filiform, 10–18 mm long; anthers deep purple, narrowly ellipsoid, 1.2–1.5 × 0.4–0.8 mm. Ovary narrowly ellipsoid, 6–10 mm long; gynophore to 5 mm long, stout. Style filiform, 9–11 mm long; stigma lobes linear. Capsules ellipsoid, 10–15 mm long, gynophore 5–10 mm long at maturity. Seeds brown, triquetrous with three narrowly winged edges, 1.1–1.5 × 0.6–0.85 mm, seedcoat coarsely reticulate.

#### Phenology.

Flowering and fruiting from December to April.

#### Distribution and habitat.

*Metagentianajiangyouensis* is currently known only from its type locality in Hanzeng Town, Jiangyou County, Mianyang City, Sichuan, China. It grows on a cliff, at an elevation between 1160 and 1513 m.

#### Etymology.

The specific epithet “jiangyouensis” refers to Jiangyou County, Mianyang City, Sichuan, China, the type locality of *Metagentianajiangyouensis*.

#### Vernacular name.

Chinese mandarin: jiang you xia rui long dan (江油狭蕊龙胆).

#### Preliminary conservation status.

*Metagentianajiangyouensis* is currently known only from its type locality in Hanzeng Town, Jiangyou County, Mianyang City, Sichuan. Based on our field survey, the habitat of *M.jiangyouensis* has been frequently disturbed by anthropogenic activities and might be compromised by touristic development. Therefore, *M.jiangyouensis* should be considered as Vulnerable (VU D2) (IUCN 2024).

#### Additional specimens examined

**(paratypes).** China • Sichuan: Mianyang City, Jiangyou County, Hanzeng Town, Qianyuan Mountain, near the Yinguangdong, growing on a cliff or on mossy rocks, 31.849795°N, 104.611639°E, 1456 m, 5 January 2019, *Yuan Zou QYS02* (paratypes: CSH!) • Same locality, 31.849402°N, 104.612286°E, 1513 m, 23 March 2024, *Hai-Feng Cao CAOHF033* (CSH!, KUN!, PE!, SMCM!) • Same locality, 1160–1180 m, 4 March 2024, *Ang Liu & Chao-Ling Yang LAJY01* (CSFI, image!).

## ﻿Discussion

*Metagentianajiangyouensis* is morphologically similar to *M.villifera* and *M.rhodantha* by the perennial habit, the presence of basal rosettes and the similar leaves and flowers. In fact, these three are the only Chinese species of the genus with basal rosettes and can therefore easily be distinguished from all other Chinese species. *M.jiangyouensis* differs from the two closely related species of *M.villifera* and *M.rhodantha* by the fact that the whole plant is glabrous, the spatulate to lanceolate calyx lobes 1.3–2(–3) mm long, the corolla tube with dark purple spots and the non-fringed plicae margin. Basal rosettes and well-developed abbreviated vegetative shoots make *M.jiangyouensis* a distinctive species amongst other species of *Metagentiana*. *Metagentianavillifera* and *M.rhodantha* sometimes have basal rosette leaves or vegetative shoots, but they are usually not well-developed or the vegetative shoots would grow into longer stems at a later time in the development. The other species of *Metagentiana* are annuals or biennials without rosettes. *M.jiangyouensis* differs from *M.rhodantha* also by its seeds with a narrow wing along the edges, whereas seeds of *M.rhodantha* are broadly winged. *Metagentianajiangyouensis* and *M.villifera* both grow on rock surfaces, but *M.rhodantha* grows in grasslands, alpine scrub, forests and on rock. *Metagentianajiangyouensis* is distributed in Jiangyou County, northeastern Sichuan, at an elevation between 1160 and 1513 m, whereas *M.villifera* is distributed in Junlian county, southeastern Sichuan, more than 420 km away from Jiangyou County, at an elevation of about 800 m; and *M.rhodantha* is widely distributed in southwest, northwest, central and south China, at an elevation between 500 and 1800 m. Furthermore, the flowering and fruiting phenophase of *M.jiangyouensis* is from December to April, while that of *M.villifera* is from April to June and that of *M.rhodantha* is from October to February. A comparison of the morphological characters of *M.jiangyouensis* and its related species is summarized in Table 1.

**Table 1.** Comparison of key characters between *Metagentianajiangyouensis*, *M.villifera* and *M.rhodantha*.

	*M.jiangyouensis*	*M.villifera*	*M.rhodantha*
Stems	8–11 cm long; green, 1–4 stems from base	20–30 cm long, purple, more than 4 stems from base	20–50 cm long, purple or green, 1–15 stems from base
Plant	glabrous	stems, leaves, and calyx densely pilose	glabrous or puberulent on petioles and leaf veins only
Basal leaves	well-developed rosette	rosette absent or present, usually poorly developed	rosette absent, if present, late growth as flowering stems
Stem leaves	glabrous, 7–12 × 4–7 mm, 6–9 pairs, margin entire	densely pilose, 5–10 × 3–9 mm, 17–21 pairs, margin entire	abaxially pubescent, 10–30 × 5–20 mm, 10–28 pairs, margin serrulate
Calyx lobes	spatulate to lanceolate, 1.3–2(–3) mm long, margin glabrous	linear-lanceolate, 3–4 mm long, margin densely pilose	linear-lanceolate, 5–10 mm long, margin ciliolate
Corolla length	2.9–3.2 cm	3–4 cm	2.5–4.5 cm
Corolla tube	with dark purple spots	without spots	without spots
Corolla lobes	4.5–6 mm long, ovate	5–7 mm long, ovate-triangular	5–9 mm long, ovate to ovate-triangular
Plicae	margin erose or denticulate, 2–3 mm long	margin fringed, 5–6 mm long	margin fringed, 4–5 mm long
Anther	1.2–1.5 mm long	2.0–2.5 mm long	2.5–3 mm long
Seeds	1.1–1.5 mm in diam., narrowly winged	__	ca. 1 mm in diam., broadly winged
Altitude	1160–1513 m	ca. 800 m	500–1800 m
Fl. and Fr.	December to April	April to June	October to February
Distribution	NE Sichuan (Jiangyou)	SE Sichuan (Junlian)	Gansu, Guangxi, Henan, W Hubei, S Shaanxi, Shanxi, Sichuan, Chongqing, Guizhou, Yunnan, Hunan, Jiangxi.

## Supplementary Material

XML Treatment for Metagentiana jiangyouensis

## ﻿Additional information

### Author ORCIDs

Hai-Feng Cao https://orcid.org/0000-0001-5489-8504

Jie Cai https://orcid.org/0000-0003-1627-3700

Yuan Zou https://orcid.org/0009-0004-8532-9207

Hong Sun https://orcid.org/0009-0006-0482-6688

Fang-Fang Li https://orcid.org/0009-0001-2404-1152

An-dong Xiong https://orcid.org/0009-0004-0959-6792

Mei-Jun Xu https://orcid.org/0009-0003-3953-4245

### Data availability

All of the data that support the findings of this study are available in the main text.
